# 贝林妥欧单抗在成人急性B淋巴细胞白血病中的应用：从诱导缓解到移植后维持的全面策略

**DOI:** 10.3760/cma.j.cn121090-20250211-00062

**Published:** 2026-02

**Authors:** 潇潇 马, 尔烈 姜

**Affiliations:** 1 天津医科大学，天津 300070 Tianjin Medical University, Tianjin 300070, China; 2 细胞生态海河实验室，天津 300020 Haihe Laboratory of Cell Ecosystem, Tianjin 300020, China; 3 中国医学科学院血液病医院（中国医学科学院血液学研究所）、血液与健康全国重点实验室、国家血液系统疾病临床医学研究中心，天津 300020 State Key Laboratory of Experimental Hematology, National Clinical Research Center for Blood Diseases, Institute of Hematology & Blood Diseases Hospital, Chinese Academy of Medical Sciences & Peking Union Medical College, Tianjin 300020, China

## Abstract

异基因造血干细胞移植（allo-HSCT）是成人复发/难治性急性淋巴细胞白血病（ALL）的重要治疗选择之一，而实现完全缓解（CR）是allo-HSCT前的关键步骤。近年来研究表明，使用双特异性T细胞衔接器（BiTE）贝林妥欧单抗进行免疫治疗，可清除残留的化疗耐药B-ALL细胞，显著提高复发/难治性B-ALL患者及微小残留病阳性患者的缓解率，成功桥接至allo-HSCT，并为移植后复发防治和维持治疗提供了更多选择。本文就贝林妥欧单抗在成人B-ALL治疗中的应用策略进行综述。

## Abstract (en)

Allogeneic hematopoietic stem cell transplantation（allo-HSCT）is an important treatment option for adult patients with relapsed/refractory acute lymphoblastic leukemia（ALL）, and achieving complete remission（CR）is a critical step before allo-HSCT. Recent studies have shown that immunotherapy with the bispecific T-cell engager（BiTE）blinatumomab can eliminate residual chemotherapy-resistant B-ALL cells, significantly improve remission rates in patients with relapsed/refractory B-ALL and those with positive minimal residual disease（MRD）, successfully bridge patients to allo-HSCT, and provide additional options for post-transplant relapse management and maintenance therapy. This article reviews the application strategies of blinatumomab in adult B-ALL.

急性淋巴细胞白血病（ALL）是淋巴样细胞在分化早期受阻所致的恶性增殖性疾病，可侵犯骨髓、外周血及髓外部位[1]。其中急性B淋巴细胞白血病（B-ALL）最为常见，占成人急性白血病的20％～30％，传统化疗虽可使完全缓解（CR）率达到70％～90％，但超过半数患者会在强化巩固期间出现复发，3～5年无病生存（DFS）率仅为30％～60％[2]。目前，异基因造血干细胞移植（allo-HSCT）仍是治愈B-ALL的关键手段，尤其适用于高危或微小残留病（MRD）持续阳性的患者，尽管儿童患者5年总生存（OS）率可达90％，成人患者的这一比例仍低于45％[3]。为预防复发，移植前后持续或新发MRD阳性患者常需接受细胞或药物干预，但往往伴随不良反应[4]。近年来，贝林妥欧单抗作为一种新型免疫疗法，能显著提高复发/难治性（R/R）B-ALL患者及CR后MRD阳性患者的缓解率与缓解深度，并有助于降低化疗强度，从而减少毒性、改善预后[5]。该药是首个获美国食品药品监督管理局（FDA）批准的双特异性T细胞衔接器（BiTE），通过激活T细胞靶向清除CD19阳性B细胞，为桥接至造血干细胞移植（HSCT）创造条件，也为移植后的维持治疗提供了新选择[6]。本文重点探讨贝林妥欧单抗在成人B-ALL患者中诱导实现CR、桥接allo-HSCT及其在移植后复发防治与维持治疗的研究进展。

一、移植前诱导巩固治疗

自FDA正式批准以来，贝林妥欧单抗已进行了多项临床试验，以验证其适用于不同人群。这些试验从治疗R/R ALL或MRD阳性的Ph阴性ALL患者开始，扩展到Ph阳性ALL患者，再到初诊B-ALL患者。多项临床试验验证了其在不同B-ALL患者人群中的疗效和安全性，为桥接移植创造了条件，并使患者长期受益。

（一）适用人群

1. R/R B-ALL患者的桥接移植优势：R/R ALL预后极差，传统化疗CR率不足30％[7]，且生存期短。多项研究表明，贝林妥欧单抗在这部分患者中展现出显著优势。TOWER试验[8]显示，贝林妥欧单抗单药治疗R/R Ph阴性ALL患者的OS期明显长于传统化疗（7.7个月对4.0个月），且获得CR的患者中MRD缓解率更高（76％对48％），整体不良反应更低（34.1％对52.3％）。ALCANTARA单臂试验证实其在Ph阳性R/R ALL患者中的有效性，CR患者的中位无复发生存（RFS）期和OS期分别为6.7个月和7.1个月，44％的患者成功桥接移植，移植后中位OS期达18个月。Apel等[9]的回顾性分析进一步支持贝林妥欧单抗可延长R/R ALL患者的生存期。

Kantarjian等[10]分析了贝林妥欧单抗作为HSCT前桥接治疗的优势，发现对于R/R ALL患者，贝林妥欧单抗治疗后桥接移植的成功率高达55％～65％，显著高于传统化疗组的30％～40％。此外，移植前MRD阴性患者的2年RFS率为62％，显著高于MRD阳性组的28％。为进一步验证贝林妥欧的疗效，Martinelli等[11]的多中心Ⅱ期临床试验纳入78例患者，结果显示CR率67％（52/78），其中MRD阴性率高达85％（44/52），62％（48/78）的患者成功接受allo-HSCT，移植后2年OS率为58％，显著优于未移植组的22％（*P*＝0.003）。这表明贝林妥欧单抗不仅能提高移植前缓解质量，还可显著改善患者长期生存。Topp等[12]的系统性综述进一步支持这一结论，报告显示接受贝林妥欧单抗桥接治疗的患者中位OS期为18.5个月，显著长于传统化疗组的9.2个月（*HR*＝0.48, *P*<0.001），接受贝林妥欧桥接治疗的患者3年OS率为58％，而单纯化疗组仅为32％（*HR*＝0.62, 95％*CI*：0.48～0.79），这进一步表明贝林妥欧为R/R B-ALL患者提供了更优的移植前准备策略。生存获益的证据也十分明确，桥接移植患者的3年OS率为58％，而未移植组仅为12％（*HR*＝0.39, *P*<0.001）。这些数据凸显了贝林妥欧单抗在移植前桥接治疗中的可选择性。

2. MRD阳性患者的移植前关键干预：MRD阳性是指初诊或R/R状态的患者经化疗、靶向治疗、嵌合抗原受体T细胞（CAR-T细胞）和（或）allo-HSCT等治疗获得血液学CR，但通过高灵敏度检测技术（如多参数流式细胞术或定量PCR）仍在血液或骨髓中检测到白血病细胞（比例≥0.01％，即≥1/10^4^个细胞）[13]。这一阈值是预测复发风险和指导治疗决策的关键指标。对于拟接受allo-HSCT的B-ALL患者，移植前实现MRD阴性是改善长期生存的核心目标[14]。贝林妥欧单抗作为BiTE，通过激活内源性T细胞靶向清除CD19阳性白血病细胞，在MRD阳性患者中效果显著。MT03-202试验[15]显示，单周期贝林妥欧单抗治疗可使87％（13/15）的Ph阳性ALL患者MRD转阴。BLAST研究[16]纳入116例强化治疗后仍MRD阳性的ALL患者，82％的患者达到MRD-CR，且MRD阴性组的OS期显著长于未缓解组（未达到对18.6个月，*P*<0.001）。成功桥接allo-HSCT的MRD阴性患者中位OS期达36.5个月，凸显了其延长生存的潜力。MRD阴性组3年RFS率和OS率分别为66％与61％，显著高于MRD阳性组的28％和32％[17]。一项真实世界研究数据（NEUF）显示，93％的Ph阴性ALL患者经贝林妥欧单抗治疗后实现MRD转阴，移植后1年非复发死亡率（NRM）仅为10.1％，显著低于传统化疗组[18]。FRENCH-CYTO研究[19]进一步证实，89％的MRD阳性患者经贝林妥欧单抗治疗后达到CR，且生存结局与基线MRD水平密切相关。这些结果表明，贝林妥欧单抗能有效清除MRD阳性患者体内的残留白血病细胞，为移植创造有利条件。

3. MRD阴性患者的巩固治疗价值：即使达到MRD阴性，采用贝林妥欧单抗巩固治疗仍可进一步改善预后。Litzow等[20]的Ⅲ期试验显示，贝林妥欧组3年OS率（85％对68％）和RFS率（80％对64％）均高于单纯化疗组，其机制可能与进一步清除隐匿性MRD及免疫微环境重塑相关（如调节性T细胞比例下调、效应T细胞功能增强）[21]。此外，巩固治疗可优化移植前疾病状态，降低移植后复发风险（*HR*＝0.58，*P*＝0.04）[16]。

（二）化疗联合贝林妥欧单抗

目前，成人ALL免疫治疗发展迅速，贝林妥欧单抗已成为一线临床试验的重要药物。研究致力于探索减少化疗毒性的替代策略，以实现化疗与贝林妥欧单抗之间的剂量平衡，并探讨其与其他免疫疗法的联合应用。一项包括7项研究的荟萃分析[22]旨在评估贝林妥欧单抗治疗前肿瘤负荷是否会影响ALL的CR率。共纳入708例R/R ALL患者，根据治疗前骨髓原始细胞百分比分为两组，结果显示骨髓原始细胞百分比<50％的患者的CR率明显高于百分比≥50％的患者（75％对33％），强调了治疗前降低肿瘤负荷的重要性。

1. 高剂量化疗联合贝林妥欧单抗：高剂量化疗Hyper-CVAD方案是ALL治疗的常用方案[23]。一项研究在Hyper-CVAD方案后序贯贝林妥欧单抗作为B-ALL一线巩固治疗，取得良好效果[24]。诱导治疗4个周期后给予贝林妥欧单抗巩固4个周期，初步报告显示，17例患者的总体反应率（ORR）为100％，MRD阴性率为93％，随访期间OS率和CR率分别为94％和93％。Nicholas等[25]研究也表明，Hyper-CVAD后序贯贝林妥欧单抗在新诊断的Ph阴性B-ALL成人患者中效果良好，34例可评估患者均获得CR，97％为MRD阴性，其中87％在第1周期后即达MRD阴性。中位随访22个月，2年RFS率和OS率分别为79％和86％，3年持续缓解率和OS率分别为80％和83％[26]。这些研究表明，高剂量化疗联合贝林妥欧单抗可提高缓解率和MRD阴性率，为移植创造更好条件。

2. 低剂量化疗联合贝林妥欧单抗：一项回顾性研究[27]纳入20例初诊B-ALL患者，采用减低剂量化疗诱导治疗后接贝林妥欧单抗诱导治疗，结果显示CR率达100％，85％（17/20）的患者实现MRD阴性。该研究成功探索了化疗和贝林妥欧单抗的剂量平衡，表明早期应用贝林妥欧单抗可减少化疗需求，为成人B-ALL诱导治疗提供了新思路。这种低剂量化疗联合贝林妥欧单抗的方案，既能降低化疗的不良反应，又能有效提高患者的缓解率和MRD阴性率，有助于提高患者对移植的耐受性，改善移植后的生存状况。

3. 化疗与贝林妥欧单抗联合奥加伊妥珠单抗（INO）：INO是一种抗体偶联药物，在R/R老年ALL患者中显示出单药活性[28]。多项研究探讨了化疗、贝林妥欧单抗与INO的联合方案。Nicholas等[29]的研究发现，在Mini-HCVD联合INO的低强度化疗方案中加入贝林妥欧单抗安全有效，总体缓解率为98％，4年OS率为50％。为评估该联合免疫疗法的安全性和有效性，Jabbour等[30]的Ⅱ期研究显示，新诊断Ph阴性B-ALL患者接受Hyper-CVAD序贯贝林妥欧单抗治疗时，添加减量INO安全且可以提高生存率。

此外，为评估贝林妥欧单抗联合化疗巩固是否优于单纯化疗巩固，ECOG-ACRIN E1910的Ⅲ期随机临床试验[31]纳入初诊成人MRD阴性（<0.01％）Ph阴性B-ALL患者。结果显示，贝林妥欧单抗联合组中位OS期未达到，而单纯化疗组为71.4个月；联合组7年OS率约为80％，单纯化疗组略高于40％，表明联合治疗显著改善生存并降低复发风险。

（三）贝林妥欧单抗实现无化疗治疗模式

研究显示，酪氨酸激酶抑制剂（TKI）联合贝林妥欧单抗的无化疗方案在一线治疗中效果显著。GIMEMA组的Ⅱ期研究[32]纳入63例新诊断的Ph阳性ALL成人患者，采用达沙替尼联合类固醇诱导治疗，随后接受2周期贝林妥欧单抗，结果显示98％的患者达到CR，中位随访18个月时，OS率和DFS率分别为95％和88％。中位随访53个月时，DFS率、OS率和无事件生存（EFS）率分别为75.8％、80.7％和74.6％，其中24例患者在首次CR期中接受了allo-HSCT[33]。MD安德森癌症中心[34]的单臂Ⅱ期试验采用普纳替尼与贝林妥欧单抗的无化疗方案，新诊断的Ph阳性ALL患者中87％达到完全分子学缓解（CMR），预估2年EFS率和OS率均为95％。国产三代TKI奥雷巴替尼联合贝林妥欧单抗治疗20例新发Ph阳性ALL患者[35]，所有患者诱导治疗后均获得CR，85％的患者在3个月内达到CMR，且无严重心血管事件或剂量调整的需求。这些无化疗方案为Ph阳性ALL患者提供了更安全有效的治疗选择，有望使部分患者后续无需移植治疗，即使需要移植，也能在移植前达到更好的状态，提高移植成功率和生存质量。

二、移植后维持与复发治疗

贝林妥欧单抗的临床试验已充分验证其有效性和安全性，目前在HSCT后的维持治疗和复发治疗中也发挥着重要作用。

（一）移植后维持治疗的生存优势

allo-HSCT是高危B-ALL巩固治疗方案，但移植相关毒性和疾病复发影响了患者的长期生存[36]。多项研究表明，贝林妥欧单抗作为移植后维持治疗具有显著优势。Gaballa等[37]的研究在高危B-ALL患者移植后第1年每3个月给予4周期贝林妥欧单抗治疗，1年OS率为85％，1年RFS率为71％，NRM率为0。Jonathan等[38]也证实移植后应用贝林妥欧单抗维持治疗可行。Nicholas等[26]的研究在维持期添加贝林妥欧单抗，使POMP维持时间从通常的3年缩短到12个月。Topp等[12]的综述数据显示，移植后接受贝林妥欧单抗维持治疗的患者2年无白血病生存（LFS）期显著长于未接受维持治疗组。我国一项基于多中心移植数据研究[39]显示，在不同供受者匹配情况下（81％的患者接受半相合供者的HSCT治疗），贝林妥欧单抗作为维持治疗展现出良好的耐受性，骨髓抑制发生率低，各亚组患者均有较好生存结局。

（二）移植后复发治疗

移植后复发患者再次缓解率低，长期生存率低，传统化疗治疗相关死亡率高[40]。多项研究显示，贝林妥欧单抗为移植后复发患者带来新希望。一项单臂Ⅱ期研究报道，64例allo-HSCT后复发患者接受贝林妥欧单抗挽救性治疗，45％的患者获得CR或部分血液学恢复（CRh）[41]。SFGM-TC的多中心回顾性研究[42]表明，贝林妥欧单抗联合供者淋巴细胞输注（DLI）治疗移植后的复发患者，CR率高于单药组（82％对50％，*P*＝0.018）。一项单中心研究显示[43]，接受贝林妥欧单抗治疗的移植后MRD阳性患者，中位缓解持续时间为12个月，且80％的患者在后续接受DLI后仍保持MRD阴性。这些数据表明，贝林妥欧单抗为移植后复发患者提供了一种有效的治疗手段，显著提高了这部分患者的缓解率，延长了缓解持续时间，为患者争取了更多的生存机会。

三、不良反应

在贝林妥欧单抗的治疗过程中，多数患者会出现一些不良事件[7]。Litzow等[20]的Ⅲ期试验显示，贝林妥欧单抗组的神经精神事件发生率显著高于化疗组（15％对5％, *P*＝0.02），包括可逆性后部脑病综合征（PRES）、癫痫和意识模糊等。其机制可能与贝林妥欧单抗介导的T细胞过度活化导致细胞因子释放（如IL-6、TNF-α）及血脑屏障通透性增加相关[44]。临床应用中需密切监测神经症状，必要时暂停治疗并给予糖皮质激素或抗癫痫药物干预[45]–[46]。

除神经毒性外，部分患者可能出现细胞因子释放综合征（CRS）、发热、乏力、感染等不良反应。临床使用时应根据患者耐受性个体化调整剂量与疗程。此外，长期或重复使用贝林妥欧单抗可能导致CD19抗原下调或丢失，从而引起免疫逃逸，影响疗效。未来需进一步探索联合用药［如与程序性死亡受体-1（PD-1）抑制剂或CAR-T细胞序贯］以克服耐药。

一项加拿大多中心研究显示，移植前接受贝林妥欧单抗治疗的B-ALL患者2年OS率更高，NRM率仅为3.2％，且Ⅱ～Ⅳ级急性移植物抗宿主病（aGVHD）发生率显著降低（27.5％对56.7％，*P*＝0.009）。表明贝林妥欧单抗在有效的同时，安全性相对可控，是目前B-ALL临床治疗的重要选择。

四、未来趋势

随着新的靶向药物的开发和现有联合方案的优化，B-ALL患者的临床预后有望进一步改善。未来关于贝林妥欧单抗的研究方向主要包括以下方面[12]。

（一）联合治疗优化

探索贝林妥欧单抗与CAR-T细胞疗法或PD-1抑制剂的序贯应用，是突破耐药瓶颈的重要方向。Webster等[47]针对R/R CD19阳性B-ALL成人患者，开展贝林妥欧单抗联合免疫检查点抑制剂（纳武利尤单抗±伊匹木单抗）的多中心Ⅰ期研究，发现联合治疗安全、有效，与MRD阴性高缓解率相关，1年OS率为63％，且接受allo-HSCT巩固治疗的患者展现出良好的长期生存前景。邓琦等[48]研究纳入5例经抗CD19 CAR-T细胞治疗后未应答或缓解后再次进展的R/R B-ALL患者，使用贝林妥欧单抗进行挽救治疗，4例（80％）在接受1～2个周期治疗后获得缓解。Chu等[49]评估了既往接受贝林妥欧单抗治疗的B-ALL患者，后续接受CAR-T细胞疗法的疗效与安全性，进一步丰富了联合治疗策略。

（二）个体化策略

根据患者遗传特征（如Ph染色体状态）动态调整贝林妥欧单抗疗程，是实现精准治疗的关键。目前相关研究正在积极推进，有望为临床实践提供更精准的指导。

（三）长期随访数据

进一步验证贝林妥欧在移植后维持治疗中提高患者长期生存率，对评估其长期疗效至关重要。尽管已有部分研究报道了贝林妥欧单抗在移植后维持治疗的短期疗效，但长期生存数据仍有待积累。如Liu等[50]的前瞻性单臂临床试验纳入计划接受allo-HSCT且移植前达到CR的B-ALL患者，使用贝林妥欧单抗作为移植后维持治疗，结果显示贝林妥欧单抗组18个月RFS率为100％，对照组为60.5％（*P*＝0.037），为后续长期随访研究提供了基础。

此外，利用二代测序技术监测超低水平MRD（灵敏度达10^−6^），实现个体化疗程调整，以及评估其长期卫生经济学优势，也值得深入研究。

五、结论

贝林妥欧单抗在HSCT患者的治疗中具有重要价值。在移植前诱导巩固治疗阶段，针对不同状态的患者（复发难治、MRD阳性、MRD阴性）均能有效提高缓解率、清除MRD，为移植创造良好条件；在移植后维持与复发治疗中，贝林妥欧单抗可降低复发风险、延长生存期，提高复发患者的缓解率。尽管存在如神经毒性、CRS及潜在免疫逃逸等不良反应，但总体安全性可控。未来随着研究的不断深入，贝林妥欧单抗有望在B-ALL治疗中发挥更大作用，进一步改善患者的预后。
